# Supplementary material for: Humoral and cellular immune responses against SARS-CoV-2 post-vaccination in immunocompetent and immunocompromised cancer populations
Source: Microbiol Spectr. 2024 Feb 14;12(3):e02050-23. doi: 10.1128/spectrum.02050-23 (PMC10913742; doi:10.1128/spectrum.02050-23)
Supplement: Figure S1 — Neutralizing antibody levels and individual T-cell responses following stimulation by Ag1, Ag2, and Ag3 in immunocompetent and immunocompromised populations. [file spectrum.02050-23-s0001.docx]

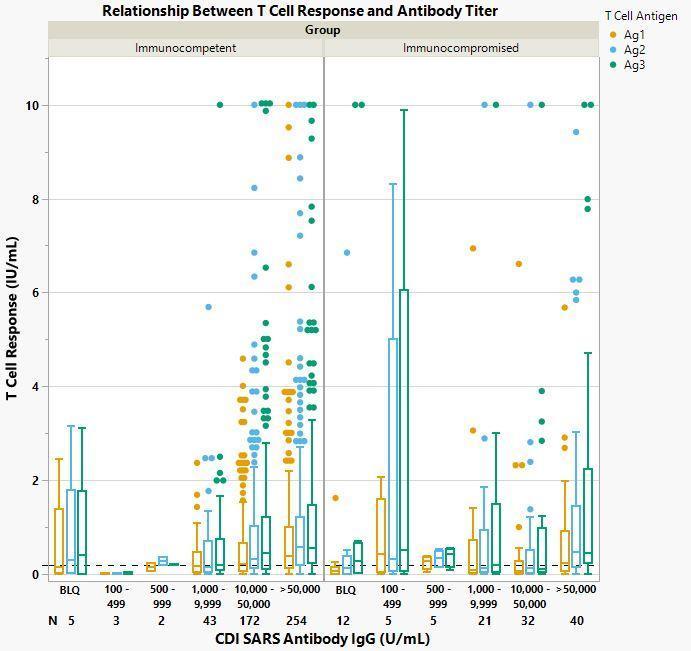


**Supplemental Figure 1**: Neutralizing antibody levels and individual T-cell responses following stimulation by Ag1, Ag2, and Ag3 in immunocompetent and immunocompromised populations.

Legend: Threshold of 0.2 IU/mL is indicated as a dashed line on the y-axis.
